# Course of activities of daily living in nursing home residents with dementia from admission to 36-month follow-up

**DOI:** 10.1186/s12877-020-01877-1

**Published:** 2020-11-20

**Authors:** Reidun Haarr Johansen, Karoline Olsen, Sverre Bergh, Jūratė Šaltytė Benth, Geir Selbæk, Anne-Sofie Helvik

**Affiliations:** 1 Department of Public Health and Nursing, Faculty of Medicine and Health Sciences, Norwegian University of Science and Technology (NTNU), Trondheim, Norway; 2 Norwegian National Advisory Unit on Ageing and Health, Vestfold Hospital Trust, Tønsberg, Norway; 3 Research Centre for Age-related Functional decline and Disease, Innlandet Hospital Trust, Ottestad, Norway; 4 Faculty of Medicine, Institute of Health and Society, University of Oslo, Oslo, Norway; 5 Institute of Clinical Medicine, University of Oslo, Oslo, Norway; 6 Health Services Research Unit, Akershus University Hospital, Lørenskog, Norway; 7 Centre for Old Age Psychiatric Research, Innlandet Hospital Trust, Ottestad, Norway; 8 Faculty of Medicine, University of Oslo, Oslo, Norway; 9 Geriatric Department, Oslo University Hospital, Oslo, Norway; 10 General Practice Research unit, Department of Public Health and Nursing, Faculty of Medicine and Health Sciences, Norwegian University of Science and Technology (NTNU), Trondheim, Norway

**Keywords:** Cognitive impairment, Functional impairment, Behavioural symptoms, Elderly, Home for the aged, Long term care, Functional decline, Psychotropic medication, PSMS, CDR

## Abstract

**Background:**

Dementia is affecting both the person with the disease and the family members. It is associated with nursing home admission, and a reduced ability to perform personal activities of daily living (P-ADL). The aim of this study was to examine the association between the severity of dementia and P-ADL function, and to study if additional factors such as neuropsychiatric symptoms, type of nursing home unit, and use of medication were associated with P-ADL function.

**Methods:**

A total of 582 nursing home residents with dementia, included at admission to the nursing home, were followed with biannual assessments for 36 months. P-ADL was assessed using the Physical Self-Maintenance scale, and severity of dementia was measured with the Clinical Dementia Rating scale. In addition, neuropsychiatric symptoms, general physical health, and use of medications were assessed at the same time points. Demographic information was collected at baseline. Linear mixed models were estimated.

**Results:**

There was a significant (*p* < 0.05) non-linear decline in P-ADL function over time in analysis not adjusting for any characteristics. More severe dementia at baseline and at the follow-up assessments was associated with lower P-ADL function (*p* < 0.001), with the association being stable over time. A higher level of neuropsychiatric symptoms, not using anti-dementia medication, being in a regular care unit as compared to a special care unit and having poor/fair general physical health as compared to good/excellent, were associated with a lower P-ADL function.

**Conclusion:**

The association between more severe dementia and lower P-ADL function was stable over a 36-month follow-up period of nursing home residents with dementia. Health care planners and clinicians should be aware of this when planning for and treating nursing home residents.

## Background

Newly published data from the United Nations (UN), state that the number of people aged 65 years or older in Europe, has increased from 8% in 1950 to 19% in 2019, and it is expected that the proportion of the elderly in the society will continue to increase [1]. The elderly population often has more comorbidities and polypharmacy [2, 3], leading to more frailty, and thus, the growing proportion of the elderly population in the society may impose a great burden to the next of kin and the health care system.

In Norway, unlike many other countries, the health care services are public [4, 5]. The primary health care service is, by law, managed by the local municipalities [6]. Services provided include social services (such as housing and home services), domiciliary care, and institutional care, mainly in nursing homes (NH). In many countries, including Norway, the NHs are institutions that provide fulltime nursing care for people when informal care and/or domiciliary care are not sufficient to fulfil the needs of care, due to the severity of illness and/or disability. Functional impairment, age, dementia, and psychosis are the main reasons for NH admission [7], with dementia being one of the factors strongest associated with admission to NH [8–10]. Approximately 80% of the long-term care residents in Norwegian NHs have dementia according to previous studies [11–13].

Dementia is a syndrome caused by a variety of brain disorders, usually of a chronic progressive nature. A progressive decline in cognitive function is one of the main characteristics of dementia. The primary risk factor for developing dementia is age, and therefore, the rising life expectancies in the society will increase the number of people who develop dementia [14]. The leading type of dementia is Alzheimer’s disease (66%) [14]. Other common causes of dementia are vascular dementia, Lewy body dementia/Parkinson disease with dementia and frontotemporal dementia [15]. According to the ICD-10 criteria for dementia, there must be a decline in memory and other cognitive abilities, such as judgement and thinking, and a decline in emotional control or motivation, without the consciousness being affected [16]. People with dementia utilize health care services more frequently [17], and dementia is not just affecting the person with the disease, but also the family members and the professional health care staff supporting the person with dementia and/or family [18–20]. Individuals with dementia often have neuropsychiatric symptoms (NPS) [21–24]. NPS include psychiatric and behavioural symptoms such as agitation, depression, hallucinations, aggression and apathy. In a descriptive study from 2018, which included the same participants as the present study, 62% of the residents with dementia had at least one clinically significant NPS at inclusion to the NH [25]. Dementia is also often associated with a reduced ability to perform activities of daily living [11, 26–31]. Several studies have found a correlation between the brain atrophy in individuals with dementia and their dysfunction in activities of daily living [32, 33]. The association between dementia and activities of daily living is demonstrated in the ICD-10 criteria for dementia, with one of the criteria being that there is a decline in cognitive abilities which causes impaired performance in everyday activities [16].

Activities of daily living (ADL) describes the ability to perform practical everyday tasks necessary for basic, and more complex self-care. It consists of instrumental ADL (I-ADL), with complex, higher order skills, and personal ADL (P-ADL), with self-maintenance skills, such as dressing, eating, bathing and toileting. A decline in P-ADL causes individuals to become more dependent of the caregivers and professional support [17, 34, 35].

The association between dementia and declined P-ADL function has been assessed in several studies and settings [11, 26–28, 31, 36–39], and some of the studies have explored the association in NH settings [11, 26, 28, 36–38]. However, most of these studies used data from various constellations of ADL-items from the mandated Minimum data set (MDS) assessment instrument used in NHs in the USA [26, 36–38]. Very few studies have used internationally accepted and validated P-ADL instruments, using a sum score of predefined aspects of P-ADL [11, 28]. Furthermore, most of the previous studies, have not adjusted for additional factors potentially important for P-ADL [11, 26, 36–38]. These factors include for example NPS, physical comorbidity, age and medication [28, 40–42].

To our knowledge, there are only two studies that have described the course of P-ADL, adjusted for variables known to have an influence on P-ADL function [28, 31]. One of the studies included older people receiving domiciliary care [31], and the other included NH residents only [28]. These studies had follow-up assessments after 18 and 12 months, respectively. More frequent assessments would give a more detailed understanding of the association between dementia and P-ADL function. Furthermore, in the study of NH residents, the residents were not included consecutively after admission to the NH [28]. To increase the understanding of the association between dementia and P-ADL function, we wanted to include the participants at the time of admission to the NH. We believed that this would give us the opportunity to explore a possible decline in P-ADL as a consequence of the NH admission, in other words, to assess whether the time after the admission had an impact on the decline in P-ADL.

The aim of this study was to examine if and how P-ADL assessed biannually over 36 months changed over time in NH residents. The primary aim was to explore the association between the severity of dementia and P-ADL function over time in NH residents. The secondary aim was to assess if and how additional factors such as neuropsychiatric symptoms, type of NH unit and use of medication were associated with the outcome.

## Methods

### Design

The data was collected from the REDIC-NH study, an observational longitudinal study including participants from a convenience sample of 47 NHs in four Norwegian counties, from both small and large NHs, located in urban and rural areas [43]. The baseline data was collected between March 2012 and November 2014, and the residents were included within one month after admission to the NH. The follow-up data was collected every six months or until the resident left the study, mainly due to death of the resident. The present study includes information from baseline (T_1_) until the 36-month follow-up (T_7_).

### Setting and participants

In total, 696 residents with an expected stay longer than four weeks were recruited at admission to the NH. Out of these, 583 had dementia [43]. All residents of 65 years and older were included, as well as residents younger than 65 years with established dementia. The only exclusion criterion was a life expectancy of less than six weeks [43]. In the present study, only those with dementia at admission, and at least one P-ADL assessment were included, resulting in 582 participants in the present study. Dementia was diagnosed at baseline according to the ICD-10 criteria independently by two physicians (SB and GS), with the possibility to consult a third physician [43]. The diagnosis was set as a research diagnosis based on all available collected data but not a clinical work-up of the patient.

### Measures

The outcome variable, personal activities of daily living (P-ADL), was assessed with the Physical Self-Maintenance Scale (PSMS) [44] in a translated Norwegian version. The scale has been frequently used in Scandinavian studies [11, 13, 28, 31], and includes six items (toileting, feeding, dressing, grooming, physical ambulation, bathing), each with five response alternatives, with a total score ranging from 6 to 30. Higher scores indicate a lower level of function.

Severity of dementia was measured with the Clinical Dementia Rating (CDR) scale. The scale covers six domains (orientation, memory, problem solving and judgment, personal care, community affairs, and home and hobbies), each with five response alternatives (0, 0.5, 1, 2, 3) [45, 46]. The CDR can be scored either according to an algorithm that gives a total score ranging from 0 (no dementia) to 3 (severe dementia) [45], or by a sum score of the six domains (CDR Sum of Boxes, CDR-SoB), ranging from 0 to 18 [47]. A higher score indicates more severe dementia. The correlation between the CDR and the CDR-SoB is high [47, 48], with the Spearman correlation of 0.87 at baseline in the original study sample [43]. Due to the increased range of values, the CDR-SoB gives important advantages over the CDR when analyzing the data [47], and in the present study the CDR-SoB was used.

Neuropsychiatric symptoms (NPS) were measured using a translated and validated Norwegian version of the Neuropsychiatric Inventory 12-item Nursing Home version (NPI-NH), including the following symptoms: delusion, hallucination, agitation/aggression, depression/dysphoria, anxiety, euphoria, apathy/indifference, disinhibition, irritability/lability, aberrant motor behavior, night-time behavior disturbances, and appetite and eating disorders [49, 50]. To obtain the item score, the severity of the symptoms (1-3) was multiplied by frequency (1-4), which provides a score from 0 to 12 on each symptom, where a higher sum indicates more severe symptoms. NPI-NH sub-syndrome scores were calculated, based on previous studies in NH residents [24, 51, 52]. The three sub-syndromes were: affective (including sum of depression and anxiety), psychosis (including sum of delusions and hallucination) and agitation (including sum of agitation/aggression, disinhibition and irritability) [24, 51, 52].

General physical health was assessed using the General Medical Health Rating (GMHR) scale, a one-item global rating scale with four response categories: poor, fair, good and excellent [53]. The scale is previously used in studies including older people with and without dementia [53–55] also in Norway [56]. For analysis, the four categories were dichotomized into poor/fair and good/excellent [25].

Use of psychotropic medications was collected from the medical record of each resident, using the Anatomic Therapeutic Chemical (ACT) Classification System. The medications were grouped into the following categories: antipsychotics (N05A except lithium), antidepressants (N06A), anxiolytics (N05B), hypnotics/sedatives (N05C), and anti-dementia medication (N06D) [57].

Demographic information such as gender, age and marital status was collected from the medical records.

### Procedure

The data collection was performed by healthcare workers in the NHs, mainly registered nurses (74%), under the supervision of 10 research nurses who had completed a five-day training program. The data collectors went through a two-day training program prior to the data collection. The training program included lessons on dementia, old-age psychiatry, geriatric assessment, and routines for data-collection, ethics, and research principles, in addition to practical training in assessment of participants. The data was collected from the medical records of each resident, and from a standardized interview with the residents, the next of kin and the residents’ caregivers in the NH.

The residents’ capacity to consent to participate in the study was considered by the NH staff, including the NH physician. A written consent for the participation was obtained from all residents who had the capacity to give consent. If a resident was lacking the capacity to give consent, their next of kin gave written consent on behalf of the resident, in line with the *Norwegian Act on medical and health research* [58]. The Regional Ethics Committee for Medical Research (REC) in South Eastern-Norway has approved the study (2011/1738a) [43].

### Data analysis

Sample characteristics at baseline were presented as means and standard deviations (SD) or frequencies and percentages.

A linear mixed model with fixed effects for nonlinear (third order) time component was estimated first to assess time trend in P-ADL-scores. Next, two models were estimated with CDR-SoB as additional fixed effect along with interaction terms between CDR-SoB and time. Model 1 included CDR-SoB assessed at baseline, while CDR-SoB measured simultaneously with P-ADL was included into Model 2. A significant interaction between CDR-SoB and time would imply a varying association between CDR-SoB and P-ADL throughout the follow-up period. Finally, Model 1 was adjusted for predefined clinical and demographic characteristics all measured at baseline, while Model 2 was adjusted for characteristics measured longitudinally whenever possible. All models included random effects for residents nested within NHs. Even though the linear mixed model allows inclusion of all available observations, also from dropouts, the dropouts may introduce bias. Therefore, baseline characteristics of dropouts and those staying in the study were compared.

All analyses were performed in SPSS version 25 and SAS version 9.4. Results with *P*-values below 0.05 were considered statistically significant. All tests were two-sided.

## Results

### Sample characteristics

The total number of participants assessed at baseline for P-ADL was 582. The mean (SD) age of these participants was 84.1 (7.5) years, ranging from 49 to 105 years. In total, 374 (64.3%) of the participants were women (Table 1). The mean baseline CDR-SoB score was 11.2 (3.6). The mean length of stay was 662 days (SD 383 days). At 36 months, 166 participants were assessed (Table 2). The main reason for leaving the study was death (*N* = 354). Those leaving the study were older (*p* = 0.001), had higher PSMS score (*p* < 0.001), higher NPI- affective sub-syndrome score (*p* = 0.017), were more often males (*p* = 0.026), and hade more often poor/fair GMHR (p < 0.001).

**Table 1:** Characteristics of study sample at baseline

Characteristics			N
**Age (years)**	Mean (SD)	84.1 (7.5)	579
**Women**	N (%)	374 (64.3)	582
**Single as marital status**	N (%)	389 (67.7)	582
**GMHR**			
Poor	N (%)	60 (10.8)	557
Fair	N (%)	220 (39.5)	557
Good	N (%)	250 (44.9)	557
Excellent	N (%)	27 (4.8)	557
**CDR-SoB**	Mean (SD)	11.2 (3.6)	575
**Type of dementia**			
AD	N (%)	414 (71.0)	582
VAD	N (%)	46 (7.9)	582
FTD	N (%)	47 (8.1)	582
LBD/PD	N (%)	22 (3.8)	582
AD/VAD	N (%)	11 (1.9)	582
Unspecified	N (%)	42 (7.2)	582
**NPI-NH**			
Agitation sub-syndrome	Mean (SD)	4.5 (7.3)	561
Psychosis sub-syndrome	Mean (SD)	1.9 (4.2)	560
Affective disorders sub-syndrome	Mean (SD)	3.9 (5.9)	572
Apathy	Mean (SD)	1.3 (2.7)	570
**Use of psychotropic medication**			
Antipsychotics	N (%)	71 (12.2)	582
Antidepressants	N (%)	167 (28.7)	582
Anxiolytics	N (%)	89 (15.3)	582
Hypnotics/Sedatives	N (%)	128 (22.0)	582
Anti-dementia medication	N (%)	163 (28.0)	582
**NH characteristics**			
RU	N (%)	367 (63.1)	582
SCU	N (%)	215 (36.9)	582

*GMHR* General Medical Health rating scale, *CDR-SoB* The sum score of the domains in the Clinical Dementia Rating scale, *AD* Alzheimer’s disease, *VAD* Vascular dementia, *FTD* Frontotemporal dementia, *LBD/PD* Lewy body dementia/Parkinson’s disease, *AD/VAD* Alzheimer’s disease mixed type, *NPI-NH* Neuropsychiatric Inventory 12-item Nursing Home version, *NH* Nursing home, *RU* Regular unit, *SCU* Special care unit

**Table 2:** Number of participants at each assessment in the study sample

	T_1_	T_2_	T_3_	T_4_	T_5_	T_6_	T_7_
Number included	582	469	387	322	269	222	171
Number assessed	582	436	372	305	260	207	166
Number left		114	82	65	53	47	51
Due to death		84	67	61	51	42	49
Other reasons		30	15	4	2	5	2
NH withdrawn		1		2			1
Resident withdrawn		4	2				
Moved to another unit of NH		13	5	2	1	5	1
Moved home		12	8		1		

T_1_: baseline, T_2_: 6-month assessment, T_3_: 12-month assessment, T_4_: 18-month assessment, T_5_: 24-month assessment, T_6_: 30-month assessment, T_7_: 36-month assessment, NH: nursing home

### Factors associated with P-ADL

**Fig. 1:**
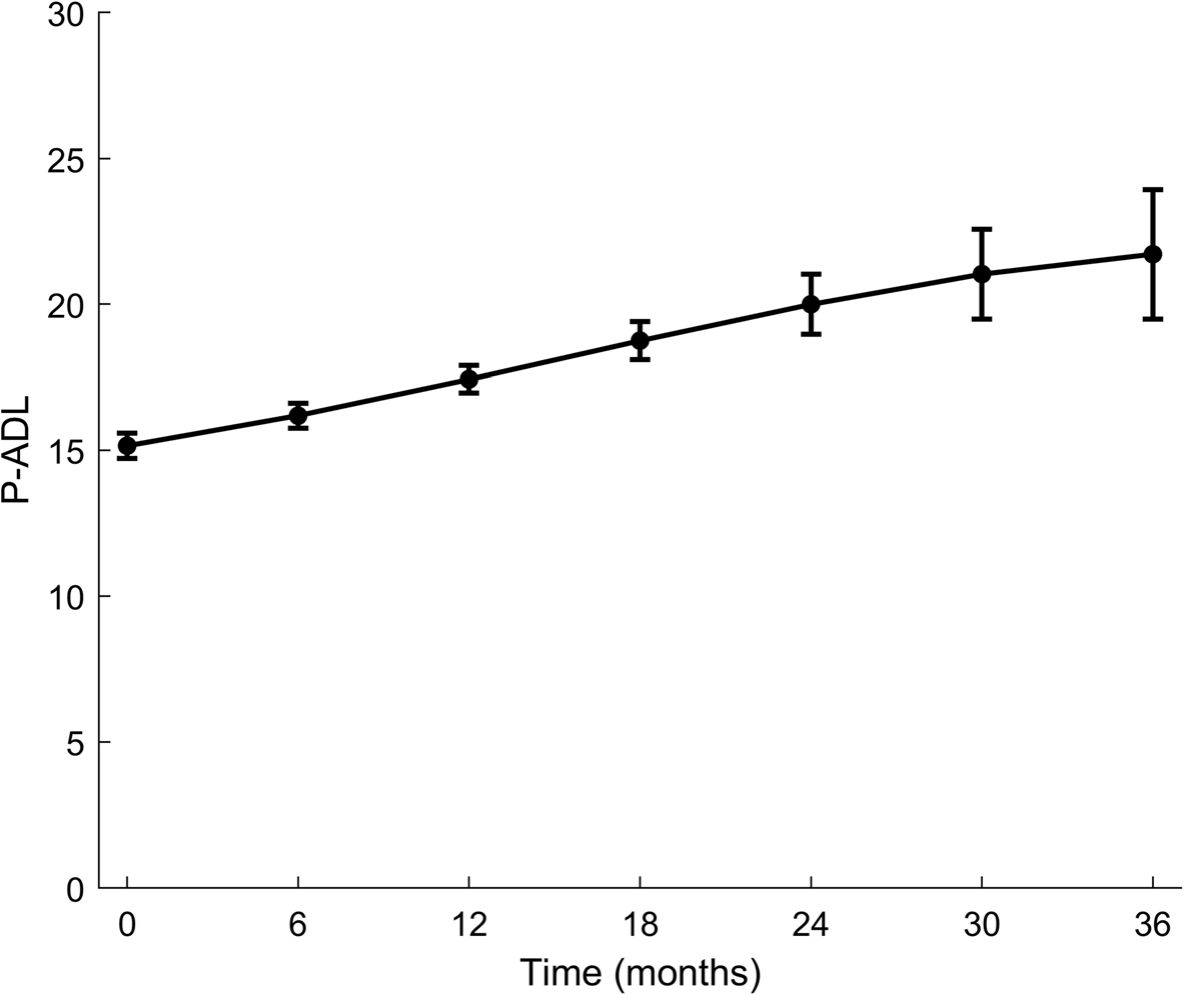
Time trend (unadjusted) in P-ADL. P-ADL: Personal Activities of Daily Living

The mean P-ADL score at baseline and at the biannual follow-up time points is presented in Table 3. At baseline the mean P-ADL score was 15.3 (4.5) and at the 36-month follow-up the score was 20.3 (4.8). There was a significant non-linear decline in P-ADL function over time in unadjusted linear mixed model (Fig. 1).

**Table 3:** P-ADL score at seven time points

Time point	Min	Max	Mean (SD)
T_1_	6	27	15.3 (4.5)
T_2_	6	27	15.9 (4.8)
T_3_	7	30	16.8 (4.7)
T_4_	8	30	17.7 (4.8)
T_5_	8	29	18.7 (4.8)
T_6_	7	29	19.7 (4.6)
T_7_	8	30	20.3 (4.8)

Higher P-ADL score indicates lower function

T_1_: baseline, T_2_: 6-month assessment, T_3_: 12-month assessment, T_4_: 18-month assessment, T_5_: 24-month assessment, T_6_: 30-month assessment, T_7_: 36-month assessment

**Fig. 2:**
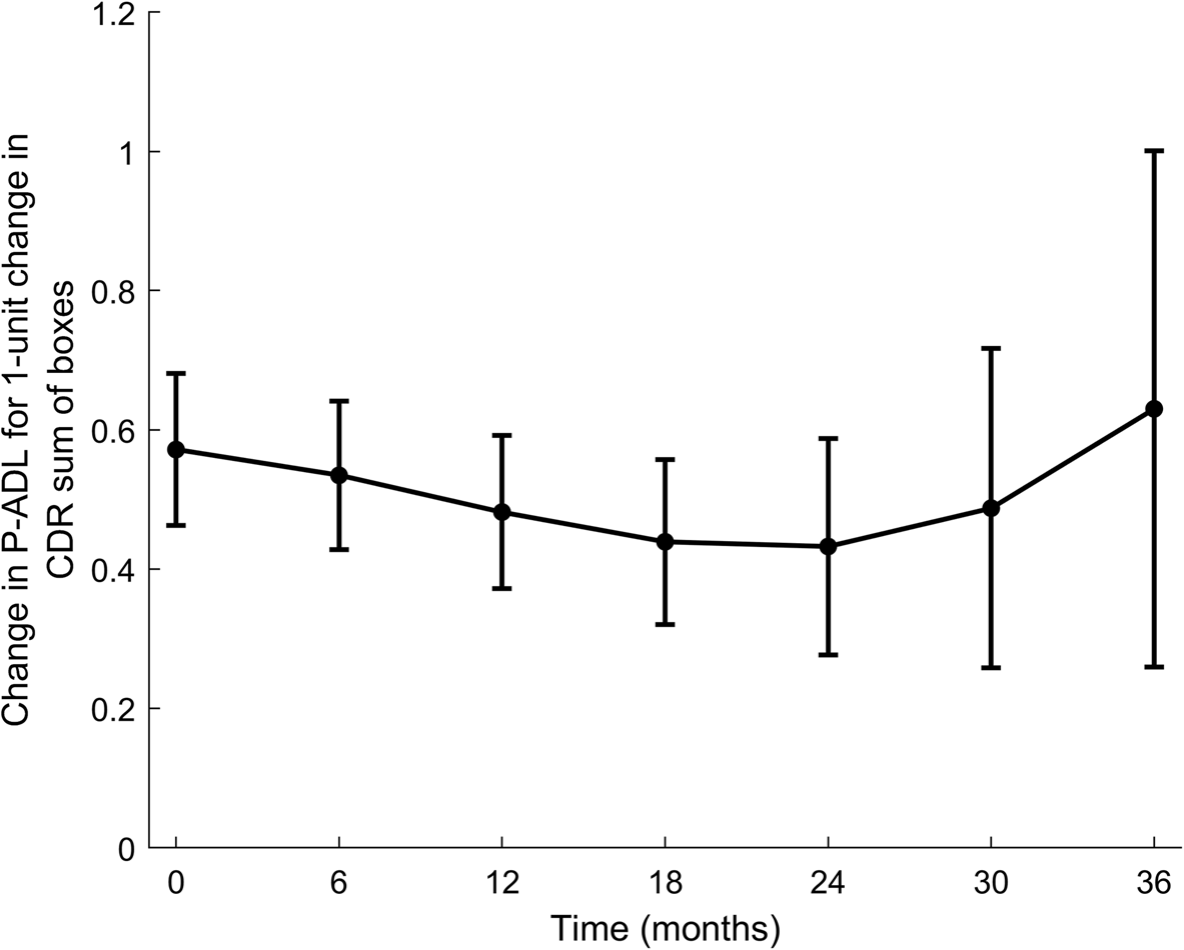
Association between P-ADL score (PSMS) and cognition (CDR-SoB) at baseline in time adjusted for covariates. PSMS: Physical Self Maintenance Scale, CDR-SoB: The sum score of the domains in the Clinical Dementia Rating scale

In both unadjusted and adjusted Model 1 (Table 4), higher baseline CDR-SoB was associated with lower P-ADL function (higher PSMS-score). This association was stable throughout the follow-up period (non-significant interaction terms), as illustrated in Fig. 2. Furthermore, in adjusted Model 1, not being single (*p* = 0.016), having poor/fair as compared to good/excellent GMHR (*p* < 0.001) and more apathy (*p* = 0.029) at baseline were associated with lower P-ADL function at each assessment.

**Table 4:** Model 1: Results of linear mixed model for effect of dementia (CDR-SoB) measured at baseline on P-ADL level over time

Covariates	Unadjusted models		Adjusted models	
	Regr. coeff. (SE)	p-value^1^	Regr. coeff. (SE)	p-value^1^
**Effect of main variable**				
Time	0.19 (0.12)	0.109	0.19 (0.12)	0.102
Time*Time	0.01 (0.01)	0.198	0.01 (0.01)	0.207
Time*Time*Time	−0.0003 (0.0002)	0.053	−0.0003 (0.0002)	0.054
CDR-SoB BL	0.59 (0.05)	**< 0.001**	0.57 (0.06)	**< 0.001**
Time*CDR-SoB BL	− 0.004 (0.01)	0.726	− 0.003 (0.01)	0.736
Time*Time*CDR-SoB BL	−0.0006 (0.0008)	0.438	−0.0006 (0.0008)	0.440
Time*Time*Time* CDR-SoB BL	0.00002 (0.00002)	0.176	0.00002 (0.00002)	0.175
**Effect of additional variables at baseline**				
*Socio –demographic information*				
Age (per years)	0.007 (0.02)	0.771	0.01 (0.02)	0.669
Women	−0.53 (0.34)	0.126	− 0.14 (0.36)	0.702
Single	−0.81 (0.36)	**0.023**	−0.91 (0.38)	**0.016**
*GMHR*				
Poor / Fair (Good/Excellent– ref.)	1.89 (0.33)	**< 0.001**	1.68 (0.33)	**< 0.001**
*NPI-NH*				
Agitation sub-syndrome	0.006 (0.02)	0.812	0.02 (0.03)	0.379
Psychosis sub-syndrome	−0.02 (0.04)	0.579	− 0.03 (0.05)	0.521
Affective sub-syndrome	−0.02 (0.03)	0.577	−0.04 (0.03)	0.231
Apathy	0.15 (0.06)	**0.020**	0.14 (0.06)	**0.029**
*Use of psychotropic medication*				
Antipsychotics	−0.38 (0.50)	0.454	−0.10 (0.51)	0.850
Antidepressants	0.14 (0.36)	0.695	0.18 (0.37)	0.631
Anxiolytics	−0.41 (0.45)	0.370	−0.46 (0.46)	0.324
Hypnotics/Sedatives	0.49 (0.39)	0.210	0.50 (0.39)	0.196
Anti-dementia medication	−0.98 (0.36)	**0.007**	−0.63 (0.36)	0.084
*NH Characteristics*				
RU (SCU – ref.)	1.06 (0.37)	**0.004**	0.51 (0.39)	0.188

CDR-SoB: The sum score of the domains in the Clinical Dementia Rating scale, BL: Baseline, GMHR: General Medical Health rating scale, NPI-NH: Neuropsychiatric Inventory 12-item Nursing Home version, NH: Nursing home, RU: Regular unit, SCU: Special care unit

*N* = 515 at T_1_, *N* = 393 at T_2_, *N* = 332 at T_3_, *N* = 271 at T_4_, *N* = 233 at T_5_, *N* = 184 at T_6_, *N* = 147 at T_7_

T_1_: baseline, T_2_: 6-month assessment, T_3_: 12-month assessment, T_4_: 18-month assessment, T_5_: 24-month assessment, T_6_: 30-month assessment, T_7_: 36-month assessment

^1^Bold text indicate *p*-value < 0.05

**Fig. 3:**
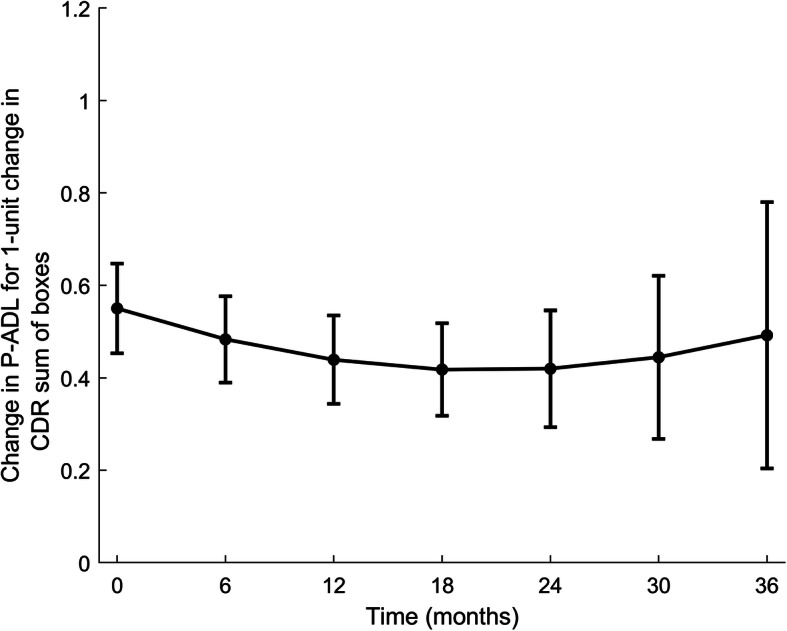
Association between P-ADL score (PSMS) and cognition (CDR-SoB) in time adjusted for covariates. PSMS: Physical Self Maintenance Scale, CDR-SoB: The sum score of the domains in the Clinical Dementia Rating scale

In unadjusted and adjusted Model 2 (Table 5), higher CDR-SoB measured simultaneously with P-ADL was associated with lower P-ADL function, with the association being stable over time (Fig. 3). In adjusted Model 2, having poor/fair as compared to good/excellent GMHR (*p* < 0.001), more apathy (p < 0.001), agitation sub-syndrome (*p* = 0.002), affective sub-syndrome (*p* = 0.001), not using anti-dementia medication (p < 0.001), and being a Regular unit (RU) resident compared to a Special care unit (SCU) resident (*p* = 0.009), all assessed simultaneously with P-ADL, were significantly associated with a lower P-ADL function.

**Table 5:** Model 2: Results of linear mixed model for effect of dementia (CDR-SoB) measured longitudinally on P-ADL level over time

Covariates	Unadjusted models		Adjusted models	
	Regr. coeff. (SE)	p-value^1^	Regr. coeff. (SE)	p-value^1^
**Effect of main variable**				
Time	0.25 (0.12)	**0.038**	0.27 (0.12)	**0.021**
Time*Time	0.005 (0.009)	0.580	− 0.0004 (0.009)	0.964
Time*Time*Time	−0.0002 (0.0002)	0.267	−0.00007 (0.0002)	0.687
CDR-SoB	0.61 (0.05)	**< 0.001**	0.55 (0.05)	**< 0.001**
Time*CDR-SoB	−0.01 (0.01)	0.242	−0.01 (0.01)	0.193
Time*Time*CDR-SoB	0.0001 (0.0008)	0.887	0.0003 (0.0008)	0.672
Time*Time*Time* CDR-SoB	0.000006 (0.00002)	0.704	−0.00000001 (0.00002)	0.996
**Effect of additional variables at baseline**				
*Socio–demographic information*				
Age (per years)	0.003 (0.02)	0.886	0.01 (0.02)	0.530
Women	−0.49 (0.33)	0.143	−0.23 (0.32)	0.476
Single	−0.65 (0.30)	**0.028**	−0.46 (0.29)	0.116
**Effect of additional variables measured longitudinally**				
*GMHR*				
Poor / Fair (Good/Excellent– ref.)	2.04 (0.16)	**< 0.001**	1.77 (0.16)	**< 0.001**
*NPI-NH*				
Agitation sub-syndrome	0.07 (0.01)	**< 0.001**	0.04 (0.01)	**0.002**
Psychosis sub-syndrome	0.07 (0.02)	**0.001**	−0.002 (0.02)	0.915
Affective sub-syndrome	0.11 (0.02)	**< 0.001**	0.06 (0.02)	**0.001**
Apathy	0.22 (0.03)	**< 0.001**	0.14 (0.03)	**< 0.001**
*Use of psychotropic medication*				
Antipsychotics	0.19 (0.26)	0.450	0.08 (0.24)	0.730
Antidepressants	−0.003 (0.21)	0.988	− 0.17 (0.20)	0.391
Anxiolytics	0.06 (0.22)	0.780	0.03 (0.21)	0.894
Hypnotics/Sedatives	0.42 (0.21)	**0.041**	0.17 (0.20)	0.382
Anti-dementia medication	−1.44 (0.22)	**< 0.001**	−1.17 (0.21)	**< 0.001**
*NH Characteristics*				
RU (vs SCU)	0.71 (0.24)	**0.003**	0.59 (0.23)	**0.009**

CDR-SoB: The sum score of the domains in the Clinical Dementia Rating scale, BL: Baseline, GMHR: General Medical Health rating scale, NPI-NH: Neuropsychiatric Inventory 12-item Nursing Home version, NH: Nursing home, RU: Regular unit, SCU: Special care unit

N = 515 at T_1_, *N* = 383 at T_2_, *N* = 320 at T_3_, *N* = 241 at T_4_, *N* = 212 at T_5_, *N* = 157 at T_6_, *N* = 121 at T_7_

T_1_: baseline, T_2_: 6-month assessment, T_3_: 12-month assessment, T_4_: 18-month assessment, T_5_: 24-month assessment, T_6_: 30-month assessment, T_7_: 36-month assessment

^1^Bold text indicate *p*-value < 0.05

## Discussion

This study of 582 NH residents with established dementia, followed with biannual assessments from admission to 36 months, showed that the degree of dementia (assessed with the CDR-SoB) at baseline, and the course of dementia at the follow-up assessments, were associated with the degree of P-ADL function. More severe dementia both at baseline and at the follow-up assessments was associated with lower P-ADL function, with the association being stable over time.

When looking at the P-ADL function adjusted for dementia assessed simultaneously with P-ADL, there was a decline in P-ADL function over time, both in unadjusted and adjusted models. This is in line with previous studies, both among NH residents and community dwelling elderly with dementia [26, 28, 31, 36–39]. However, none of these studies assessing older adults with dementia in NHs included the residents consecutively after admission to the NH. By including the residents at the time of admission, as was done in the present study, the changes that might occur due to the time spent in the NH, will be the same for all the participants, and thus the study sample will be more homogenous when assessed over time.

Many studies have shown that more severe dementia is associated with lower P-ADL function in NH residents [26, 28, 36–38]. In the present study, we also found that the association between P-ADL function and degree of dementia was stable over time. In a 52-month follow-up study of NH residents from Norway, the rate of decline in P-ADL function explained by the degree of dementia, decreased during follow-up [28], which differs from the present study. The follow-up time in that study was considerably longer than in the present study, and it is not clear whether a 52-month follow-up with biannual assessments of the present study would show the same results. Furthermore, the NH study mentioned above had few follow-ups (3 in total), and the NH residents were not included consecutively after admission to the NH. This may possibly explain the differences in the results between the 52-month study and the present study.

It could be questioned whether the P-ADL of an individual with dementia under 65 years could be compared to the P-ADL of an older person with dementia. Our clinical experience is that those being admitted to a Norwegian nursing home with dementia are in a state that makes municipal domiciliary care insufficient, due to dementia, P-ADL function and/or NPS, independent of age. With the PSMS scale assessing very basic activities of daily living, the measured P-ADL are comparable even if the age range of those admitted to the nursing home are broad. Nevertheless, age adjustments in the analysis should be performed, as was done in this study. However, in the present nursing home study where all participants were included at admission to the nursing home, we did not find an association between age and P-ADL or decline in P-ADL. In line with this finding, a Japanese study of persons with dementia living at home found no difference in P-ADL decline in those of 75 years or older compared to younger people [59]. However, a previous Norwegian Nursing home study from 2015 found higher age associated with lower P-ADL function [31].

In several previous studies, both among participants with and without dementia, NPS have been found to be associated with P-ADL function [28, 31, 40, 41, 60–62]. In the present study, we found that more apathy symptoms, and higher agitation and affective sub-syndrome scores were associated with a lower P-ADL function. Even though we found an association between NPS-syndromes and P-ADL function, it remains unclear whether the worsening of NPS gives a lower P-ADL function, or if it is the other way around. However, an explanation for these results, can be that there is a common underlying reason, that gives both more NPS, and a lower P-ADL function. Several structural changes in the brain have been associated with problems in P-ADL function [32, 33, 63]. Changes in some of the same areas have been associated with having NPS [64]. This suggests that there could be common underlying structural changes in the brain that lead to both more NPS and problems in P-ADL function.

In the present study, the use of anti-dementia medication was associated with a better P-ADL function, which is in line with previous studies [28, 31, 34, 65]. This association remained significant even when controlling for additional factors, suggesting that clinicians may consider using these medications with the intention of improving the residents’ P-ADL function, and not only their cognitive function. It is well known that there are a variety of structural changes in the brain in the different types of dementia. Studies have shown that anti-dementia medication can delay these structural changes [66]. Many of the same areas are affected in individuals with a low P-ADL function, and our results could thereby be explained by the fact that anti-dementia medication also delays the changes in the brain associated with the loss of P-ADL function. To our knowledge, however, this association has not yet been explored, and further research is therefore required to explore the impact anti-dementia medication has on the brain, as well as on P-ADL function. However, another explanation for the results in the present study, can be that the residents with the lowest P-ADL function may not be able to utilize the possibly low effect of the anti-dementia medication. Thus, it may be a lower indication for giving these residents anti-dementia medication, and there will therefore be fewer people with low P-ADL function among the residents who uses these medications.

As expected, there was an association between P-ADL function and GMHR. In addition, it was found that belonging to a SCU at a NH was associated with better P-ADL function, compared to those who belonged to a RU. We do not know if the reasons for getting these results are due to differences in the structure and staffing between RU and SCU, or other factors, such as rest confounding.

### Strengths and limitations of the study

One of the main strengths of the present study, is that all the participants were included at the time they were admitted to the NH, and they were followed with biannual assessments for a long follow-up time (36 months). Furthermore, the study used several validated measuring tools, such as PSMS for P-ADL and NPI-NH for NPS. In addition, the results in the present study were adjusted for variables known to have an influence on P-ADL function, in contrast to many previous similar studies [11, 26, 36, 37].

Despite the strengths of the study, there are also limitations. The main weakness in this study is many dropouts, mostly due to death, leading to fewer participants and less data collected. To accommodate any degree of imbalance in the data, a linear mixed model was used. This model is well-suited for analysing data with missing values and dropouts, by including all available data. However, those leaving the study were older and had poorer functioning and physical health than those participating. These findings may imply some degree of bias, but they also indicate that the decline in P-ADL might be underestimated rather than overestimated.

Due to the prospective design of the study, it was necessary to include all NH residents when they were admitted to a NH. In order to restrict time of inclusion, the study included new residents at several NHs over a large geographical area. We consider this as a strength, because it gives a better representativity than including only a few NHs over a smaller geographical area. On the other hand, it also led to many research coordinators and data collectors involved in the study. This may have caused some differences in interpreting the assessment protocol and tools, which might have reduced the data validity. To secure as high validity of the data as possible, the research coordinators completed a five-day training program, and the data collectors completed a two-day training program, prior to the data collection. Despite this, we cannot rule out the possibility of differences in the data collection.

It is also worth mentioning the proxy reporting of P-ADL and symptoms as a potential source for bias, however, it is recommended that P-ADL functioning is based in observation by care-taker in individuals with more severe cognitive impairment [41, 67].

Even though the study adjusted for many variables relevant for P-ADL function, the analysis did not include information about education. Some previous studies have shown that educational background has an impact on P-ADL function [68–70]. However, to our knowledge, there is only one study that has explored this association among people with dementia [28], and this study did not find education to have a significant impact on P-ADL. Thus, since the importance of education for the association between severity of dementia and P-ADL in NH residents is still uncertain, we cannot exclude the possibility that information about education could have influenced the estimates in the present study.

## Conclusion

This 36-months longitudinally study of NH residents with dementia, followed with biannual assessments (*N* = 582 at baseline, and *N* = 166 at 36-months), found that more severe dementia at baseline and at the follow-up assessments was associated with lower P-ADL function. This association was stable over time. The health care planners and clinicians should therefore pay attention to degree of dementia, and other factors associated with P-ADL function in NH residents, in order to improve the quality of care for the residents with dementia, and to help maintain their level of function as long as possible.

## Data Availability

The datasets generated and/or analysed during the current study are available for researchers in cooperation with the data owner, the Research Centre for Age-related Functional decline and Disease, Innlandet Hospital Trust. Information is available on the following page link: https://sykehuset-innlandet.no/avdelinger/alderspsykiatrisk-avdeling/forskningssenteret-for-aldersrelatert-funksjonssvikt-og-sykdom.

## Abbreviations

NH: Nursing home
RU: Regular unit
SCU: Special care unit
NPS: Neuropsychiatric symptoms
ADL: Activities of daily living
I-ADL: Instrumental activities of daily living
P-ADL: Personal activities of daily living
PSMS: Physical Self-Maintenance Scale
MDS: Minimum Data Set
BL: Baseline
T1: baseline
T2: 6-month assessment
T3: 12-month assessment
T4: 18-month assessment
T5: 24-month assessment
T6: 30-month assessment
T7: 36-month assessment
CDR: Clinical Dementia Rating
CDR-SoB: The sum score of the domains in the Clinical Dementia Rating scale
NPI-NH: Neuropsychiatric Inventory 12-item Nursing Home version
GMHR: General Medical Health rating scale
ATC: Anatomic Therapeutic Chemical
REC: Regional Ethics Committee for Medical Research
SD: Standard Deviations
AD: Alzheimer’s disease
VAD: Vascular dementia
FTD: Frontotemporal dementia
LBD/PD: Lewy body dementia/Parkinson’s disease
AD/VAD: Alzheimer’s disease mixed type
